# Relationship Between Patient Portal Tool Use and Medication Adherence and Viral Load Among Patients Living with HIV

**DOI:** 10.1007/s11606-023-08474-z

**Published:** 2024-01-22

**Authors:** Amanda M. Midboe, Shayna Cave, Stephanie L. Shimada, Ashley C. Griffin, Tigran Avoundjian, Steven M. Asch, Allen L. Gifford, Donald Keith McInnes, Lara K. Troszak

**Affiliations:** 1 VA HSR&D Center for Innovation to Implementation (Ci2i), VA Palo Alto Health Care System, Menlo Park, CA USA; 2 Department of Public Health Sciences, School of Medicine, University of California, Davis, CA USA; 3 Center for Healthcare Organization and Implementation Research, Veterans Affairs Bedford Healthcare System, Bedford, MA USA; 4 Department of Health Law, Policy, and Management, Boston University School of Public Health, Boston, MA USA; 5 Department of Population and Quantitative Health Sciences, UMass Chan Medical School, Worcester, MA USA; 6 Stanford University School of Medicine, Stanford, CA USA; 7 Division of Primary Care and Population Health, Stanford University School of Medicine, Stanford, CA USA; 8 Section of General Internal Medicine, Boston University School of Medicine, Boston, MA USA

## Abstract

**Background:**

Patient portals play an increasingly critical role in engaging patients in their health care. They have the potential to significantly impact the health of those living with chronic diseases, such as HIV, for whom consistent care engagement is both critical and complex.

**Objective:**

The primary aim was to examine the longitudinal relationships between individual portal tool use and health-related outcomes in patients living with HIV.

**Design:**

Retrospective cohort study using electronic health record data to examine the relationship between patient portal tool use and key HIV-specific, health-related outcomes in patients engaged in care in the Veterans Health Administration (VA) through the application of marginal structural models.

**Participants:**

A national sample of patients living with HIV (PLWH) active in VA care who were registered to use the VA’s patient portal, My Health*e*Vet (MHV; *n* = 18,390) between 10/1/2012 and 4/1/2017.

**Main Measures:**

The MHV tools examined were prescription refill (including prescription refill of an antiretroviral (ART) medication and any medication), secure messaging, view appointments, and view labs. Primary outcomes were viral load test receipt, viral load suppression, and ART medication adherence (measured as proportion of days covered).

**Key Results:**

The use of prescription refill for any medication or for ART was positively associated with ART adherence. Secure messaging was positively associated with ART adherence but not with viral load test receipt or viral load suppression. The use of view appointments was positively associated with ART adherence and viral load test receipt but not viral load suppression. The use of view labs was positively associated with viral load suppression but not ART adherence or viral load test receipt.

**Conclusions:**

These findings highlight the valuable role patient portals may play in improving health-related outcomes among PLWH and have implications for patients living with other types of chronic disease.

**Supplementary Information:**

The online version contains supplementary material available at 10.1007/s11606-023-08474-z.

## INTRODUCTION

With the rapid, federally subsidized spread of electronic health records that came with the United States (US) Health Information Technology for Economic and Clinical Health (HITECH) Act of 2009, there has been a growing interest in patient portals—secure, web-based platforms for sharing healthcare information with the patient, managed by the healthcare organization.^1^ Stage 2 of the HITECH act, which began in 2014, was an important facilitator for the expansion and use of patient portals.^2^ This stage focused on improving health information exchange between patients and providers and increasing patient access to their health information through portals. In the US, patient portals have evolved in scope of use, with approximately 90% of healthcare systems offering portals with varying functionalities.^3^ In recognition of the importance of portals in chronic disease management, the Chronic Care Model was revised to include eHealth,^4^ which highlights the role of portals in increasing patient’s knowledge, self-efficacy, and skills for health self-management.

A growing body of research shows that patient portals have the potential to improve health-related outcomes, particularly for individuals living with chronic disease.^5^ Patients report they are convenient and provide useful information.^6^ They also find portals valuable to facilitate engagement in their care through communicating and coordinating with their care teams.^7^ Aside from the benefits patients perceive, a growing body of research has shown that use of specific portal tools is positively related to certain health-related outcomes. Among patients living with diabetes, more months of portal use was related to improved hemoglobin A1c (HbA1c) and low-density lipoproteins control,^8^ with earlier research showing that sustained use of the secure messaging tool among patients was related to controlled HbA1c among those who had uncontrolled HbA1c at baseline.^9^ Similarly, in a sample of patients living with HIV (PLWH), use of the prescription refill tool was associated with changes in viral load from unsuppressed to suppressed over time.^10^ Among PLWH living in the Southeastern US, registration to use a patient portal was linked with retention in care and viral suppression, but unfortunately evaluation of specific tool use was not examined.^11^

The present study addresses gaps in the previous literature by examining the longitudinal relationship between specific portal tool use and health-related outcomes in a national sample of PLWH. We relied on a marginal structural modeling approach, which is a type of causal inference analysis with longitudinal data that provides adjustments for time-varying confounding in observational studies such as this one.^12^ The portal tools examined include prescription refill (herein referred to as Rx refill) for any medication and for antiretroviral (ART) medications specifically, secure messaging, view labs, and view appointments. In line with previous work, the health-related outcomes included were medication adherence, viral load testing, and viral suppression.^10, 11^ Given some extant literature has included a focus on those living with HIV who were not virally suppressed at time of using the portal, an additional exploratory analysis was also completed.

The Veterans Health Administration (VA) is the ideal setting for this study. It is the largest integrated healthcare system in the US and was one of the first healthcare organizations to have a patient portal. In 2003, the VA launched My Health*e*Vet (MHV), which now has more than 5 million registered users.^8, 13^ The functionality of MHV has expanded over time, enhancing the usability and adding tools that promote self-management.^14^ Patients engaged in VA care who are living with HIV have shown some of the highest rates of MHV adoption,^15^ and recent research shows that 49% of these patients have registered for MHV.^16^

Given the primary aim of this study was to examine the relationships between specific portal tool use and health-related outcomes in PLWH, we opted not to extend the observed cohort into the COVID-19 pandemic time-period. Healthcare as usual changed significantly, including for those living with HIV. McGinnis and colleagues^17^ found that that viral load testing dropped significantly throughout 2020 for PLWH in VA, which impacts two primary outcomes of this study—viral load testing receipt and viral suppression. Nevertheless, in an attempt to understand how use of the patient portal (MHV) may have changed during COVID-19 in the cohort examined in this study, we summarize portal use across our study period and 2 years after the COVID-19 pandemic began.

## METHODS

We selected an initial cohort of PLWH (i.e., one inpatient or two outpatient encounters for HIV during fiscal years (FY) 2011 to 2017). Fiscal years begin on October 1 and end on September 30 the following year (e.g., FY11 is October 1, 2010, to September 30, 2011). We then identified those who had at least registered to use MHV between October 1, 2012, and April 1, 2017. During this period, two types of MHV accounts were available to all patients, an *Advanced* account and a *Premium* account. When they registered, they automatically received an *Advanced* account which provided access to Rx refill. To gain access to the other tools examined in this study, MHV registrants had to authenticate their identity, through in-person authentication (IPA), which some patients did not do. Due to variation in access to MHV tools depending on account type, we utilized different subsets of our cohort of MHV registrants to evaluate the impacts of Rx refill on outcomes versus the impacts of all other tools. Additionally, our observation of MHV engagement was restricted to FY13 and later; FY13 was the first FY that MHV data were available for research.

For analyses examining Rx refill, we included all MHV registrants who registered for MHV between the aforementioned time-period (i.e., “refill access cohort”). For analyses examining all other tools, we included all MHV registrants who IPAed during the same time-period (i.e., “full access cohort”). By defining these cohorts according to these dates, we initiate our observation of patients at the time when the MHV tool became accessible to them. We therefore reduce selection bias by adjusting for baseline measurements taken immediately prior to their choice to gain access to the MHV tool. These groups are not mutually exclusive, nor is one group a direct subset of the other. Also, given the longitudinal structure of our study, a patient could be included in one or both cohorts for different lengths of time. For example, a PLWH who registered for MHV on October 13, 2012 and IPAed on August 5, 2014 would belong to only the refill access cohort for several intervals but would belong to both cohorts for all intervals following their IPA date.

Data were obtained primarily from VA electronic health record (Corporate Data Warehouse). Additional sources of data were used to measure housing status and geographic characteristics, and they are described in greater detail in prior work.^13^ We observed patients from their MHV registration (or IPA) date until September 30, 2018, or until their death if before the end of the study period. Institutional Review Board (IRB) approval was obtained from the Stanford University IRB.

### Data Structure

The impacts of MHV tool use on outcomes were assessed in 6-month intervals, according to the VA FY. Whereas we began observing PLWHs on their registration date (or IPA date), patient-time did not contribute to estimation of the outcome models until we had observed (1) required baseline measurements in the six months prior to their registration date, (2) at least two full intervals of MHV registrant status (required to obtain the values for MHV tool use and 6-month lagged MHV tool use), and (3) the last full intervals of outcome observation occurred after the interval in which MHV tool use was measured. The figure in Appendix 1 depicts an example of the temporal order in which covariates and outcomes were measured using the example of outcome measurement in the second half of FY14.

#### Exposures

The following indicators were examined in a given 6-month interval as the exposures of interest: (1) any Rx refill use, (2) Rx Refill use to refill an ART, (3) secure messaging use, (4) view appointments use, and (5) view labs use. For each interval, we designate these indicators as 1 if a PLWH used the tool at least once, and as 0 if they did not use the tool.

#### Time-invariant Covariates

All of these covariates were measured before the patient observation entry data (except non-VA care): age, race/ethnicity, sex, FY half of cohort entry, and years since the PLWH’s first VA recorded HIV diagnosis. Measurement details can be found in prior work.^13, 16^ Non-VA care was included as a fixed indicator of non-VA care that was paid for by the VA at any point in the study period.

#### Time-varying Covariates

Measures of time-varying covariates were taken in each 6-month interval from a PLWH’s entry into observation until their death or the end of the study period. These covariates include ZIP code-level residence rurality, ZIP code-level residence Area Deprivation Index, housing status, number of outpatient visits, number of outpatient visits related to HIV, number of inpatient stays, primary Veterans Integrated Service Network (VISN) of care, the Elixhauser Comorbidity Index, and ICD-10 codes for depression, bipolar disorder, psychoses, post-traumatic stress disorder (PTSD), substance use disorder (SUD), and alcohol use disorder (AUD). Additional details are provided in prior work.^13^

#### Missing Data

PLWH who did not have access to MHV tools beyond Rx refill during a specific time period were assigned zeros for other MHV tools in that wave. In the case of a missing area deprivation index measure for the current time period, baseline, or lag 6 months, an average of all time intervals was taken and imputed. PLWH missing this measure for all time intervals were excluded from the cohort. Missing race/ethnicity were assigned to an “unknown” category, which was then pooled with American Indian or Alaska Native, Native Hawaiian or Other Pacific Islander, and Asian to form the “Unknown/Other” race/ethnicity category. PLWH missing rurality information were assigned to an “unknown” category for that time period. PLWH missing baseline VISN information were excluded.

### Primary Outcomes

#### Medication Adherence

Proportion of Days Covered (PDC) is the percentage of days a PLWH theoretically has their medication based on VA pharmacy data. It is the preferred medication adherence measure of the Pharmacy Quality Alliance and used in previous studies of ART.^18–20^ An ART regimen includes at least 2 classes of drugs that minimize viral resistance. To be counted as adherent, a PLWH must have 2 or more classes of ARTs on any given day and prescribed from FY12 to FY18. Each medication and respective class of drug can be found in Appendix 2 as well as additional details for calculating adherence.^21^ Adherence was treated as a continuous variable with values ranging from 0 to 1 (vs a binary variable). Adherence from the 6 months after MHV tool use was assessed in models to better judge temporal effects.

#### Viral Load Testing and Suppression

VA laboratory data was used to find cohort instances of viral load testing and whether those labs resulted in suppressed or unsuppressed viral load, specifically nucleic acid–based quantitative viral load testing. Additional details for metric refinement are in Appendix 3. In each 6-month period, the most recent test was chosen as the viral load suppression value of interest. PLWH were considered to have received a viral load test in a 6-month period if any test result in that time had an interpretable numeric value. For viral load suppression, a last-observation-forward approach was used. Both viral load suppression and viral load test receipt were treated as binary variables. The viral load test receipt from the 6 months after MHV tool use was assessed in the model to better judge temporal effects. The denominator for viral load suppression includes those who had a baseline viral load test; they stay in the cohort if they meet other criteria, including VA activity and no record of death.

### Statistical Analyses

Longitudinal marginal structural models, using inverse probability of treatment weighting (IPTW), were used to examine the relationship between MHV tool use and the primary outcomes.^22^ These models can address possible selection bias, loss-to-follow-up, and complex time-varying confounding, making them a highly useful approach in this instance.^23^ Appendix 3 provides details about this modeling approach. The outcome models were estimated using IPTW-weighted generalized estimating equations. Specifically, we estimate the effects of MHV tool use on adherence, viral load suppression, and viral load test receipt, conditional on baseline and time-fixed covariates. Robust standard errors, an autoregressive correlation structure of order one, clustering by patient, and fixed effects for baseline VISN were employed (see Appendix 4 for R packages used). As noted by Althouse (2016)^24^, corrections for multiple comparison may obscure relevant findings in an exploratory study such as this one; confidence intervals are provided to support interpretation of our findings.

## RESULTS

Among 34,606 PLWH and active in VA healthcare between 10/1/2012 and 4/1/2017, there were 18,390 PLWH who were registered users of MHV (53.1%). These PLWH were broken up into two cohorts—refill access and full access cohorts. There is a significant overlap of patients represented in these cohorts, ranging from 56.4 to 73.9% throughout the intervals in the study period.

### Refill Access Cohort

Drawing from 18,390 patients, this cohort included 8102 (44.1%) who registered; 3823 (20.8%) with required baseline adherence; 3757 (20.4%) with required baseline VISN; and 3749 (20.4%) with a ZIP code, making up a final person time amount of 17,596 6-month periods. An additional 189 patients were excluded due to missing baseline viral load, for a total of 3560 (19.04%) patients and 16,655 6-month periods.

### Full Access Cohort

**Fig. 1:**
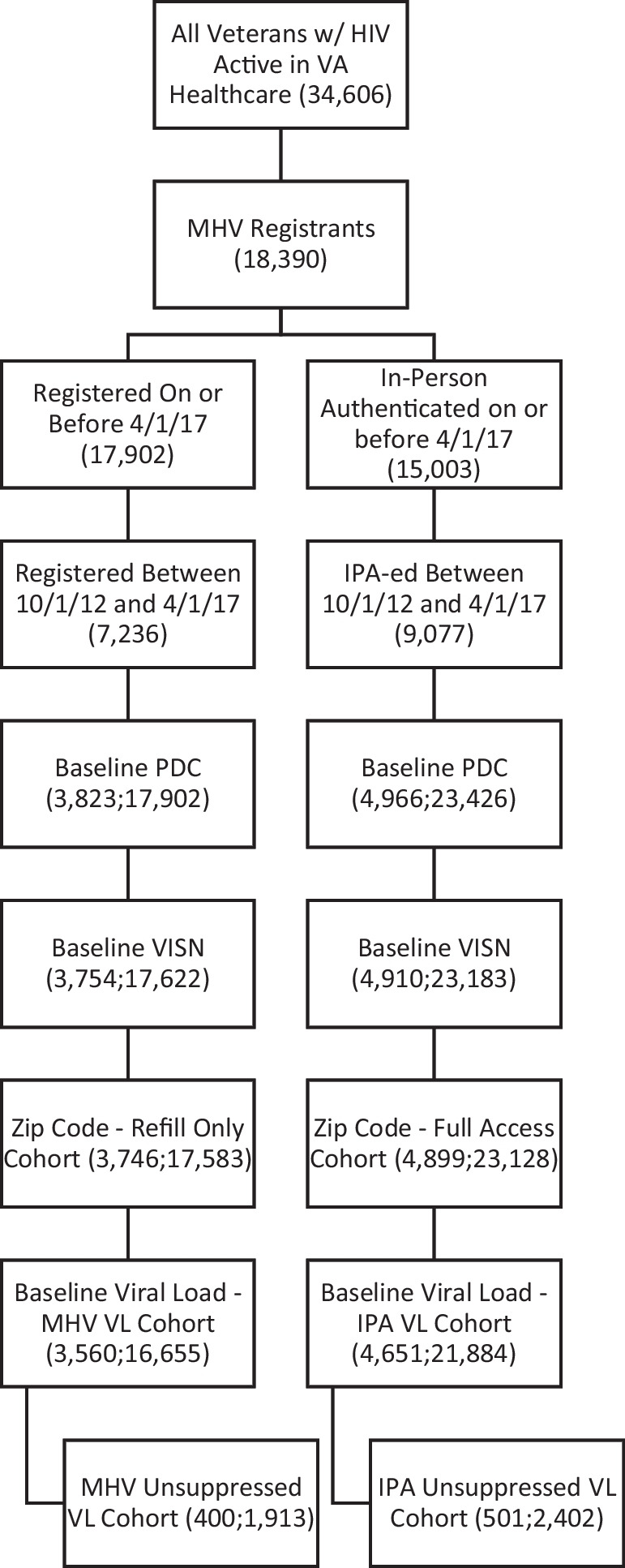
**CONSORT Diagram. Note. Refill access cohort: Of the 18,390 PLWH, 8102 (44.1%) registered between 10/1/12 and 4/1/17; 3823 (20.8%) had the required baseline PDC (adherence); 3757 (20.4%) had the required baseline VISN; and 3749 (20.4%) had a zip code at some point during the cohort, making up our final person time amount of 17,596 6-month intervals. For the viral load cohorts, an additional 189 PLWHs were excluded due to missing baseline viral load. Full access cohort: Of the 18,390 PLWH, 9077 (49.4%) IPA-ed between 10/1/12 and 4/1/17; 4966 (27.0%) had the required baseline PDC (adherence); 4910 (26.7%) had the required baseline VISN; and 4899 (26.6%) had a zip code at some point during the cohort, making up our final person time count of 23,128 6-month intervals. For the viral load cohorts, an additional 248 PLWH were excluded due to missing baseline viral load.**

Drawing from 18,390 patients, this cohort included 9077 (49.4%) who IPA-ed; 4966 (27.0%) with required baseline PDC; 4910 (26.7%) with required baseline VISN; and 4899 (26.6%) with a ZIP code, making up a final person time count of 23,128 6-month periods. An additional 248 patients were excluded due to missing baseline viral load, for a total of 4651 (25.3%) patients and 21,884 6-month periods (see Fig. 1).

Table 1 provides baseline demographic and covariate data for the refill access (*n* = 3823) and full access (*n* = 4,966) cohort, respectively. To determine if those who had a baseline adherence measure differed significantly from those who were missing a baseline adherence, we examined key demographic measures (i.e., sex, race/ethnicity, age). There were no significant differences in the two groups. Table 2 provides a summary of percent of patient time with individual MHV tools and average outcomes by tool use for the refill access and full access cohorts, respectively. Table 3 provides a summary of all MHV model outcome results.

**Table 1:** Baseline Demographics and Characteristics of PLWH Registered for MHV or IPA-ed Between FY13-FY18 in Person-Time with Baseline ART Adherence

Cohort	Refill access	Full access
Number of observations	17,902	23,426
Unique patients	3823	4966
Categorical data	n (%)	n (%)
Sex		
Female	643 (3.6)	809 (3.5)
Male	17,259 (96.0)	22,617 (97.0)
Race/ethnicity		
Black or African American	8988 (50.0)	10,526 (45.0)
Hispanic or Latino	1356 (7.6)	1773 (7.6)
White	6577 (37.0)	9860 (42.0)
Other or unknown	981 (5.5)	1267 (5.4)
Baseline rural/urban status		
Rural	2714 (15.0)	3819 (16.0)
Urban	14,528 (81.0)	18,883 (81.0)
Unknown	660 (3.7)	724 (3.1)
Numerical data	Mean (SD)	Mean (SD)
Number of 6-month time periods	4.7 (1.7)	4.7 (1.7)
Age	54 (11)	53 (11)
Time since diagnosis (days)	3284 (1736)	3300 (1703)
Time since diagnosis (years)	9.0 (4.8)	9.0 (4.7)
Any non-VA visits during cohort	0.31 (0.46)	0.32 (0.47)
Baseline measures		
Medication adherence	0.81 (0.39)	0.83 (0.38)
Outpatient any visits	13.3 (15.5)	12.8 (14.6)
Outpatient HIV visits	2.9 (3.2	2.8 (3.0)
Inpatient any stays	0.18 (0.61)	0.16 (0.56)
Elixhauser	1.6 (1.7)	1.6 (1.6)
Housed	0.86 (0.35)	0.88 (0.32)
Area deprivation index	54.1 (21.0)	52.9 (20.9)
Alcohol use disorder	0.12 (0.32)	0.10 (0.31)
Bipolar	0.04 (0.21)	0.05 (0.22)
Depression	0.29 (0.45)	0.29 (0.45)
Psychoses	0.04 (0.20)	0.04 (0.19)
Posttraumatic stress disorder	0.11 (0.31)	0.11 (0.31)
Substance use disorder	0.15 (0.36)	0.13 (0.34)

**Table 2:** Summary of Patient-Time and Outcomes by MHV Tool Use

Cohort	MHV tool	Outcome variable	Patients represented	Percent patient time using tool	Patient time using tool	Total patient time	Average outcome in time periods with tool use	Average outcome in time periods without tool use
Refill access	Rx refill for ART	PDC	3746	18.5%	3247	17,583	0.87	0.75
Refill access	Rx refill use	PDC	3746	21.2%	3720	17,583	0.85	0.75
Full access	Secure messaging	PDC	4899	18.0%	4166	23,128	0.86	0.76
Full access	View appointments	PDC	4899	27.8%	6438	23,128	0.85	0.76
Full access	View labs	PDC	4899	18.6%	4305	23,128	0.85	0.77
Refill access	Rx refill for ART	Viral load suppression	3560	18.6%	3099	16,655	95%	91%
Refill access	Rx refill use	Viral load suppression	3560	21.3%	3544	16,655	94%	91%
Full access	Secure messaging	Viral load suppression	4651	18.3%	3999	21,884	95%	91%
Full access	View appointments	Viral load suppression	4651	28.1%	6160	21,884	94%	91%
Full access	View labs	Viral load suppression	4651	18.7%	4086	21,884	95%	91%
Refill access	Rx refill for ART	Viral load test receipt	3560	18.6%	3099	16,655	84%	79%
Refill access	Rx refill use	Viral load test receipt	3560	21.3%	3544	16,655	83%	79%
Full access	Secure messaging	Viral load test receipt	4651	18.3%	3999	21,884	84%	79%
Full access	View appointments	Viral load test receipt	4651	28.1%	6160	21,884	84%	78%
Full access	View labs	Viral load test receipt	4651	18.7%	4086	21,884	83%	79%
Unsuppressed	Rx refill for ART	Viral load suppression	400	15.2%	291	1913	77%	65%
Unsuppressed	Rx refill use	Viral load suppression	400	17.3%	330	1913	76%	65%
Unsuppressed	Secure messaging	Viral load suppression	501	12.1%	291	2402	75%	67%
Unsuppressed	View appointments	Viral load suppression	501	24.8%	596	2402	72%	66%
Unsuppressed	View labs	Viral load suppression	501	16.1%	387	2402	76%	66%

Refill access = PLWH who at least have access to the Rx refill tool; full access = PLWH who have access to all tools. *Finding is significant at the α = 0.05 level. The numerator for the percent patient time using the tool is count of unique days using the tool within a given interval for each unique patient

**Table 3:** All MHV Tools and Outcomes Model Results, 1st/99th Percentile Truncated and Normalized

Cohort	Tool of interest	Patients represented	Estimate	Confidence interval
Outcome: adherence				
Refill access	Rx refill for ART	3746	0.0212*	(0.0079, 0.0345)
Refill access	Rx refill use	3746	0.0202*	(0.0055, 0.0349)
Full access	Secure messaging	4899	0.0148*	(0.0025, 0.0271)
Full access	View appointments	4899	0.0107*	(0.0020, 0.0194)
Full access	View labs	4899	0.0045	(−0.0055, 0.0145)
Outcome: viral load suppression				
Refill access	Rx refill for ART	3560	1.2202	(0.9402, 1.5836)
Refill access	Rx refill use	3560	1.2573	(0.9764, 1.6190)
Full access	Secure messaging	4651	0.8896	(0.7157, 1.1058)
Full access	View appointments	4651	0.9402	(0.7733, 1.1431)
Full access	View labs	4651	1.2943*	(1.0032, 1.6700)
Outcome: viral load test receipt				
Refill access	Rx refill for ART	3560	1.1027	(0.9282, 1.3101)
Refill access	Rx refill use	3560	1.0633	(0.8839, 1.2792)
Full access	Secure messaging	4651	1.1252	(0.9531, 1.3284)
Full access	View appointments	4651	1.1877*	(1.0545, 1.3377)
Full access	View labs	4651	0.8772	(0.7572, 1.0163)

Refill access = PLWH who at least have access to the access Rx refill tool; full access = PLWH who have access to all tools. *Finding is significant at the α = 0.05 level

### Medication Adherence

Most MHV tools were found to be positively associated with PDC (adherence): Rx refill for ART had a coefficient of 0.0212 (C.I. 0.0079, 0.0345), Rx refill had a coefficient of 0.0202 (C.I. 0.0055, 0.0349), secure messaging had a coefficient of 0.0148 (C.I. 0.0025, 0.0271), and view appointments had a coefficient of 0.0107 (C.I. 0.0020, 0.0194). Compared to their respective non-MHV tool users, these results mean that, on average, Rx refill for ART users have an estimated 2.12% increase, Rx refill users have an estimated 2.02% increase, secure messaging users have an estimated 1.48% increase, and view appointments users have an estimated 1.07% increase in expected PDC (adherence) in the 6 months following that tool’s use.

### Viral Test Receipt and Load Suppression

View appointments was positively associated with viral load test receipt (1.1877, C.I. 1.055, 1.338) and view labs was positively associated with viral load suppression (1.2943, C.I. 1.0032, 1.6700). When compared to non-MHV tool users with the same baseline characteristics and time spent in the cohort in the 6 months following that tool’s use, MHV users of view labs and view appointments experience an increase in odds ratio of an estimated 19% of receiving a viral load test and 29% of being virally suppressed.

### Exploratory Analysis of an Unsuppressed Viral Load Cohort

The exploratory unsuppressed viral load MHV cohort limited the cohort to 400 (2.2%) PLWH and 1913 time periods, with an unsuppressed viral load full access cohort of 501 (2.7%) PLWH and 2402 time periods. These PLWH also had lower overall viral load suppression compared to the larger cohort during all time points in both tool-use time-periods (72–77% v. 94–95%) and no-tool-use time-periods (65–67% v. 91%; Table 4).

**Table 4:** Exploratory Unsuppressed Viral Load Subgroup Model Results, 1st/99th Percentile Truncated and Normalized

Cohort	Tool of interest	Outcome of interest	Patients	Total patient-time	Estimate	Confidence interval
Refill access unsuppressed	Rx refill for ART	Viral load suppression	400	1913	0.8114	(0.5366, 1.227)
Refill access unsuppressed	Rx refill use	Viral load suppression	400	1913	0.9164	(0.6567, 1.2788)
Full access unsuppressed	Secure messaging	Viral load suppression	501	2402	0.6603*	(0.4613, 0.9452)
Full access unsuppressed	View appointments	Viral load suppression	501	2402	0.9913	(0.7638, 1.2865)
Full access unsuppressed	View labs	Viral load suppression	501	2402	1.5667*	(1.1184, 2.1949)

Refill access = PLWH who at least have access to the Rx refill tool; full access = PLWH who have access to all tools. *Finding is significant at the α = 0.05 level

In these subgroups, View Labs was again positively associated with viral load suppression (1.5667, C.I. 1.1184, 2.1949), resulting in an increase in odds ratio of an estimated 57% of being virally suppressed when compared to non-MHV tool users. Secure messaging was negatively associated with viral load suppression (0.6603, C.I 0.4613, 0.9452), resulting in a decrease in odds ratio of an estimated 34% of being virally suppressed when compared to non-MHV tool users with the same baseline characteristics and time spent in the cohort in the 6 months following that tool’s use.

### Trends in MHV Tool Use

**Fig. 2:**
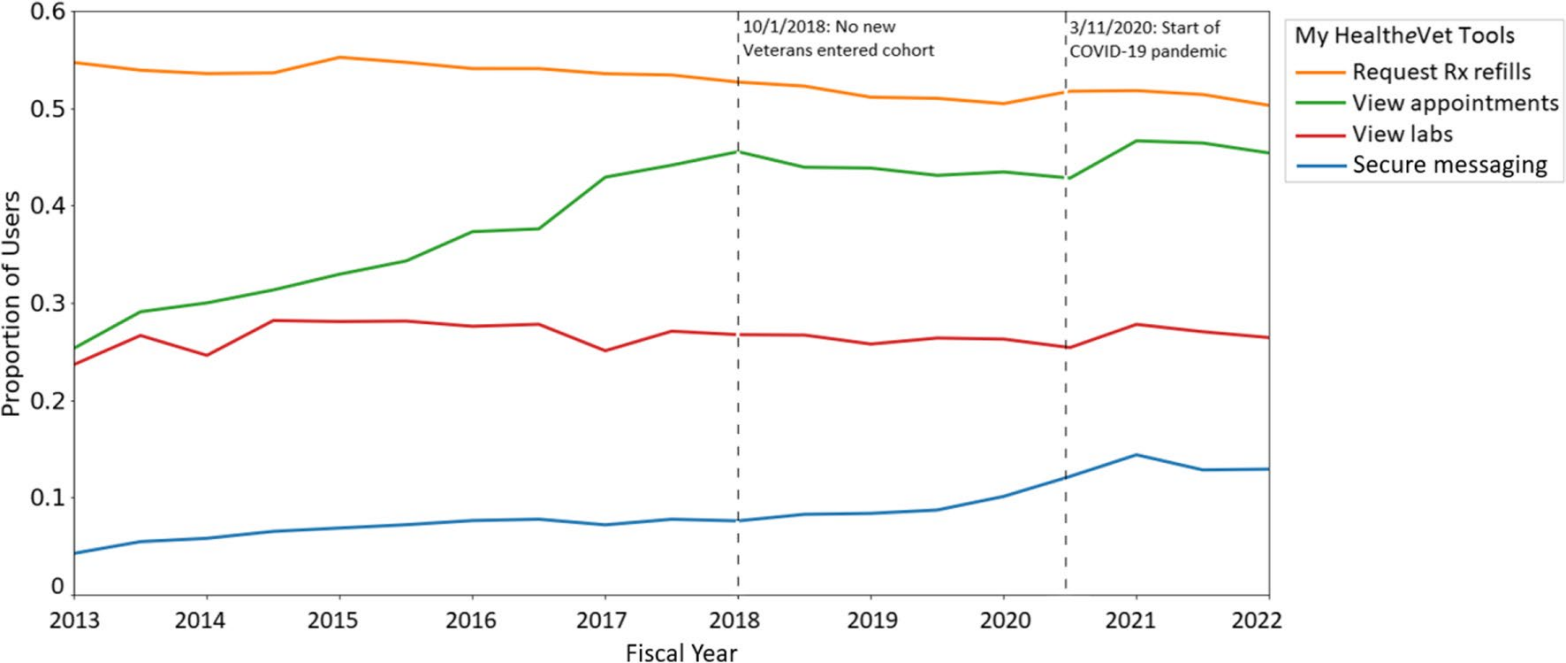
**My Health***e*Vet tool use over time (10/1/2012–3/25/2022). Note. Each feature proportion is calculated in 6-month periods by: the number of distinct PLWH who used the feature on at least two separate days within a year divided by the number of PLWH with a diagnosis of HIV who were alive and had registered for MHV.

Among PLWH continuously engaged in care (i.e., had a VA appointment in the last two years, have a future appointment, or have a primary care panel), the proportion of PLWH viewing appointments and sending secure messages increased over the last decade (Fig. 2). Viewing lab results and requesting Rx refills remained relatively steady during this period, with a slight decline in the proportion of PLWH requesting Rx refills.

## DISCUSSION

This study adds to the growing literature on patient portal use and health-related outcomes among PLWH^10, 11^ and has implications for those living with chronic disease. To our knowledge, this is the first comprehensive longitudinal examination of use of specific portal tools and health-related outcomes in a national sample of patients living with HIV. We found that use of view appointments was positively associated with viral load test receipt. Importantly, most portal tools examined in this study were positively associated with ART medication adherence, with the exception of view labs. However, the use of view labs was positively associated with viral load suppression, which we also found as part of our exploratory analysis, which compared those with viral suppression to those without viral suppression.

Although secure messaging has been positively associated with health outcomes among patients living with diabetes,^8^ we did see not see a consistent benefit of secure messaging across all health outcomes. Secure messaging was positively associated with medication adherence but not viral load suppression or viral load test receipt. Exploratory analysis of those who were not virally suppressed revealed a negative association between secure message use and viral suppression. Future research that examines the relationship between content of secure messages and health-related outcomes is needed, particularly in light of previous work, which has found that secure message content can vary significantly across users.^25^ The null findings showing no association between prescription refill and viral load suppression should also be investigated in future research given previous findings showing a relationship between MHV refill use and viral load in a smaller sample of PLWH.^10^

There are steps that healthcare organizations can take to improve access and use of patient portals, including focused outreach to patients living with HIV who have had significant barriers to access, including Black patients^16^ as well as those from vulnerable populations (e.g., homeless, living with a substance use disorder).^13^ Lack of provider encouragement of portal use may account for some of these disparities, with a recent study showing providers were less likely to encourage portal use among Black and Hispanic individuals.^26^

To improve equitable access to patient portals, there are key strategies that healthcare organizations can implement. For example, a VA study found that distributing tablets to those with access barriers increased registration among non-users and increased portal use among those already registered.^27^ Healthcare organizations can also focus efforts on provider education about the benefits of portal use by patients, as revealed by a recent qualitative study of patients living with HIV and their providers.^28^ Providers also reported needing more education about how portals can benefit patients, and patients reported wanting providers to encourage their use.^28^

Although there are key strengths of this study, as mentioned previously, there are some important limitations to mention. The ideal study design to show impact of patient portal use on health-related outcomes would be a randomized control trial, but that was not possible, particularly given that VA’s patient portal has been available to users since 2003. We attempted to control for threats to validity through the use of longitudinal marginal structural models, but future work should consider approaches in design phase of observational studies to mitigate the possibility of certain types of bias, such as collider bias.^29^ We did not adjust for multiple comparisons given the exploratory nature of this study. In future studies, the testing of associations from a priori hypotheses derived from this study should consider correction for multiple comparisons.^24^ Lastly, a potential limitation is not examining the period during the COVID-19 pandemic, which saw an unprecedented expansion of telehealth modalities.^30^ These have been challenging to assess until recently. However, now that more than 3 years have elapsed since the initiation of the COVID pandemic, healthcare has begun to settle into a new normal, with significant disruptions to healthcare abating. As such, an important area for future research will be to examine how pivoting to telehealth and increasing reliance on other virtual modalities, such as patient portals, may impact the health of those living with chronic disease.

### Supplementary Information

Below is the link to the electronic supplementary material.

Supplementary file1 (DOCX 46 KB)
