# Supplementary material for: Relationship Between Patient Portal Tool Use and Medication Adherence and Viral Load Among Patients Living with HIV
Source: J Gen Intern Med. 2024 Jan 22;39(Suppl 1):127–35. doi: 10.1007/s11606-023-08474-z (PMC10937883; doi:10.1007/s11606-023-08474-z)
Supplement: Supplementary file 1 — Supplementary file1 (DOCX 46 KB) [file 11606_2023_8474_MOESM1_ESM.docx]

Appendix 1. Temporal Order of Measurement Example
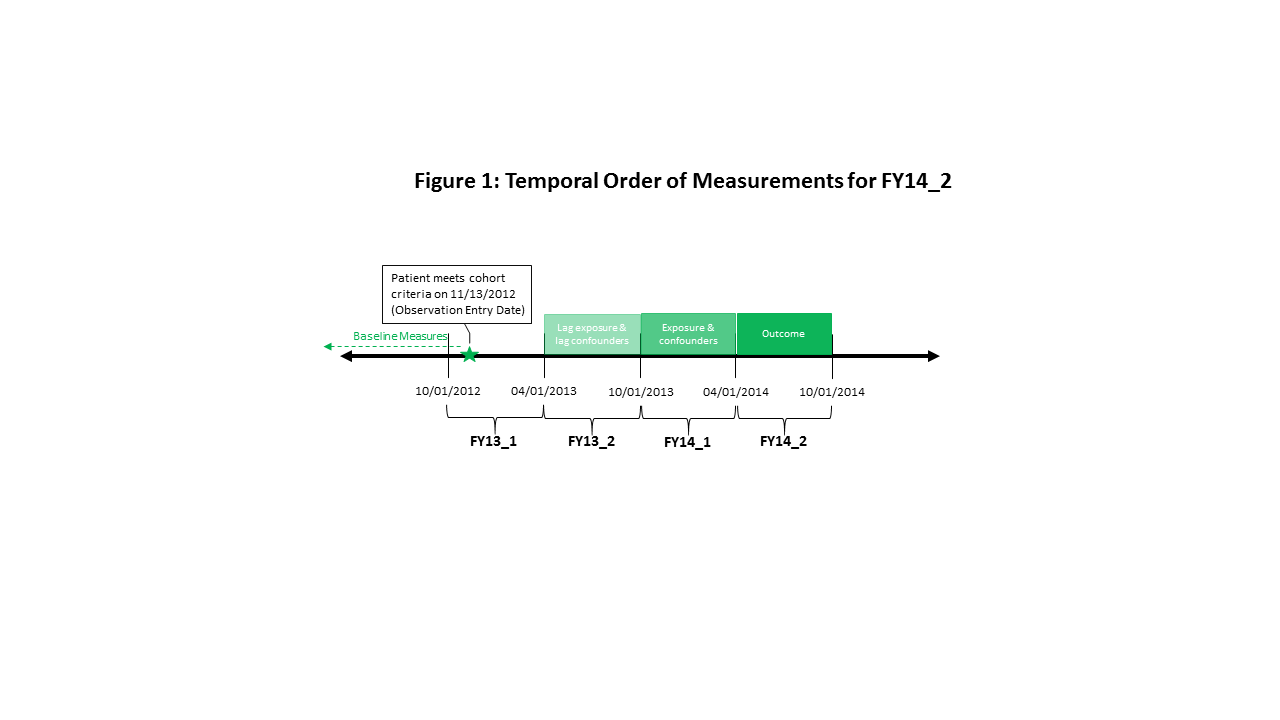


Appendix 2. HIV Medications and Class Memberships

| **Entry Inhibitors** | | | | | |
| --- | --- | --- | --- | --- | --- |
| Enfuvirtide | Maraviroc |  |  |  |  |
| **Integrase Inhibitors** | | | | | |
| Bictegravir | Dolutegravir | Elvitegravir | Raltegravir |  |  |
| **Non-Nucleoside Reverse Transcriptase Inhibitor** | | | | | |
| Delavirdine | Doravirine | Efavirenz | Etravirine | Nevirapine | Rilpivirine |
| **Nucleoside Reverse Transcriptase Inhibitor** | | | | | |
| Abacavir | Didanosine | Emtricitabine | Lamivudine | Stavudine (d4t) | Tenofovir |
| Zidovudine |  |  |  |  |  |
| **Protease Inhibitors** | | | | | |
| Atazanavir | Darunavir | Fosamprenavir | Indinavir | Lopinavir | Nelfinavir |
| Saquinavir | Tipranavir |  |  |  |  |
| **Combination** | | | | | |
| Abacavir/Dolutegravir/Lamivudine | | | Bictegravir/Emtricitabine/Tenofovir | | |
| Dolutegravir/Rilpivirine | |  | Efavirenz/Emtricitabine/Tenofovir | | |
| Emtricitabine/Rilpivirine/Tenofovir | | | Cobicistat/Darunavir/Emtricitabine/Tenofovir | | |

Ritonavir and cobicistat alone were excluded from these data, as these two drugs are primarily or exclusively (respectively) used as protease inhibitor boosters, with either serious side effects or no ART drug activity (respectively) when used alone.

Additional PDC methods

From the first prescription date to either the cohort end in FY18 or a patient’s death date, each drug received an indicator of coverage for each day. Within the same class of ARTs, coverage by multiple drugs was only counted as one class. Each day was considered adherent if it contained two or more distinct classes. The total number of days adherent were then broken up into FY 6-month periods and divided by the number of days a patient spent in that time period to obtain PDC. When a prescription of the same drug had days of overlap with its corresponding previous prescription, the new prescription’s start date is pushed forward by the number of overlapping days in order to account for extra supply. The final prescription’s end day was considered final regardless of overlap or extra supply. One additional week of prescriptions in FY19 was included in our data due to account for disproportionately low PDC in the second half of FY18.

**Additional Viral Load Testing and Viral Load Suppression Methods**

Results with identifiable numeric values received that number, and results that stated the patient’s HIV was not detected or under the detectable limit received half of the stated detectable limit or 100 copies/mL if the limit was not specified. If the value was below 8, the result was assumed to be a log value and was exponentiated with base ten. Values of 200 copies/mL or more were considered unsuppressed HIV, whereas values under 200 were considered suppressed. In the rare case where a patient had both values equal to or above and below 200 on the same day, the average of the non-log values was chosen if available, then the average of the log values, then the average of values extracted from comments.

Appendix 3. Marginal Structure Models and Measurement of Medication Adherence and Viral Load

Marginal structural models first require construction of inverse probability of treatment weights (IPTW). Particularly in MSMs that examine longitudinal exposures and outcomes, IPTWs can exhibit extreme variability, resulting in the possibility that a very small number of individuals/time periods with extreme weights determine most of the estimated impact of the exposure on the outcome (Robins et al., 2000). To address this, we first employed weight stabilization.

Weight stabilization is a standard practice used in the construction of IPTW for longitudinal MSMs. This strategy adds greater specificity and balance to the weights by comparing a patient only to those with similar baseline and time-fixed values. It also weakens the required positivity assumption by only requiring there to be both exposed and unexposed veterans within all strata of time-varying covariates that exist within each level of the baseline and time-fixed covariates (Cole et al., 2008).

For each patient, i, and at each time point, t, stabilized weight s were constructed as follows:

$$sw_{it}=\frac{f_{A_{it}|V_{i}}\left( A_{i,t},V_{i} \right)}{f_{A_{it}|L_{it,t-1},A_{i,t-1}}(A_{i,t,t-1},V_{i},L_{i,t,t-1})}$$

where $f\left( \cdot\right)$ is a probability density function, $A_{i,t}$ is the exposure status of patient i at time t, $A_{i,t-1}$ is the exposure status of patient i at time t-1 (value in the previous 6 months), $V_{i}$ is the time-invariant covariate profile for patient i, $L_{i,t}$ is the time-varying covariate profile for patient i at time t, and $L_{i,t-1}$ is the time-varying covariate profile for patient i at time t-1 (values in the previous 6 months).

In practice, this requires constructing separate numerator and denominator exposure prediction models. Lag indicates the previous 6-month’s values in the example models below.

**Numerator model (example for Rx Refill/PDC):**

Rx Refill ~ FY Half of Registration + Race/Ethnicity + Age at Entry + Sex + Time Since HIV Diagnosis at Entry + Non-VA Indicator + Baseline Outpatient Visits + Baseline HIV Outpatient + Baseline Inpatient + Baseline Rurality + Baseline ADI + Baseline Housing Status + Baseline Elixhauser + Baseline SUD + Baseline AUD + Baseline Psychoses + Baseline Depression + Baseline Bipolar + Baseline PTSD + Baseline VISN + Baseline PDC

**Denominator model (example for Rx Refill/PDC):**

Rx Refill ~ lag(Rx Refill) + FY Half of Registration + Race/Ethnicity + Age at Entry + Sex + Time Since HIV Diagnosis at Entry + Non-VA Indictaor + Baseline Outpatient Visits + Baseline HIV Outpatient + Baseline Inpatient + Baseline Rurality + Baseline ADI + Baseline Housing Status + Baseline Elixhauser + Baseline SUD + Baseline AUD + Baseline Psychoses + Baseline Depression + Baseline Bipolar + Baseline PTSD + Basline VISN + Baseline PDC + Secure Messaging + View Appointments + View Labs + Outpatient Visits + HIV Outpatient + Inpatatient + Rurality + ADI + Housing Status + Elixhauser + SUD + AUD + Psychoses + Depression + Bipolar + PTSD + VISN + lag(Secure Messaging) + lag(View Appointments) + lag(View Labs) + lag(Outpatient Visits) + lag(HIV Outpatient) + lag(Inpat) + lag(Rurality) + lag(ADI) + lag(Housing Status) + lag(Elixhauser) + lag(SUD) + lag(AUD) + lag(Psychoses) + lag(Depression) + lag(Bipolar) + lag(PTSD) + lag(VISN) + lag(PDC)

To minimize assumptions about the data-generating process that gave rise to MHV tool use in our study population, we employed a stacked-ensemble method, Super Learner (Van der Laan et al, 2007), to estimate both the numerator and denominator models contributing to the IPTW. This approach allows for a combination of candidate models, both parametric and non-parametric, to contribute to the estimation of the IPTW. The resulting weight models are as asymptotically close to correctly specified as is possible given the candidate models. We included the following models in the Super Learners: empirical mean, binomial generalized linear model (GLM), binomial GLM with restricted cubic splines, random forest, and xgboost boosted trees.

Ridge regression was excluded due to its use of inner fold cross-validation (CV) for selection of lambda, which could have led to patients predicting their own past and future timepoints. Lasso was excluded due to the desire to keep all parameters included in the model rather than shrinking some to zero. We chose to use restricted cubic splines, which can be implemented within a GLM, rather than smoothing splines, which require the use of GAMs and in some cases, inner-fold CV to choose the best smoothing parameter.

Within the binomial GLM with splines, only the numerator model received spline options, as the denominator model already had too many variables for the required degrees of freedom. Age, time since diagnosis at entry, baseline outpatient visits, baseline outpatient visits for HIV, baseline Elixhauser, Baseline ADI, and Baseline PDC (when applicable) all received cubic splines with a model for 3 internal knots and a model for 4 internal knots each, locations chosen using percentiles.

For random forest, the models were given parameter options based on the assumption that approximately the square root of the number of predictors will result in the best performance. The numerator received options for the floor of 0.5, 0.75, 1, and 2 times the square root of the number of predictors, while the denominator received options for the floor of 0.5, 1, 1.25, 1.5, 1.75, and 2 times the square root of the number of predictors. From preliminary data runs, the numerator appeared to benefit from fewer predictors whereas the denominator benefited from more predictors. All options used 1000 trees.

For xgboost, parameters were chosen ahead of time to avoid using inner cross validation. The numerator could handle smaller shrinkage parameters due to having fewer predictors, which resulted in lower depth requirements. 12 xgboost model varieties were tried: 100 trees, shrinkage 0.1, depth 1-3; 1000 trees, shrinkage 0.1, depth 1-3; 1000 trees, shrinkage 0.01, depth 1-3; 5000 trees, shrinkage 0.001, depth 1-3. The denominator required more depth due to having more predictors, resulting in higher shrinkage to reduce runtime and computation requirements. 13 xgboost model varieties were tried: 100 trees, shrinkage 0.1, depth 1-5; 1000 trees, shrinkage 0.01, depth 1-5; 5000 trees, shrinkage 0.01, depth 1-3.

All person-times were included in a single numerator and a single denominator model for each combination of exposures and outcomes to increase data available and to decrease overall run time. The predicted numerator probabilities were divided by their respective denominator probabilities to obtain initial weights, then multiplied for each patient up to including the correct time point to obtain the marginal structural model weights accurate to each time point.

Weight truncation was utilized to minimize the effects of the most extreme weights by restricting the highest and lowest weights to a chosen weight percentile; we chose to truncate at the 1^st^ and 99^th^ percentiles for all models. This adds a small amount of bias in exchange for a decrease in variance in the estimation of our target parameter. Practically, this prevents patients with enormous weights from dominating the model and allows a more comprehensive distribution of data points to inform estimation (Cole et al., 2008).

Finally, normalization was utilized to ensure weights had an average of one. When weights have a mean greater than one or less than one, the sample is artificially inflated or deflated in size, resulting in incorrect standard errors and thus, incorrect inference. To combat this, each weight was divided by its respective time period’s average to achieve a normalized distribution ([Xiao 2010](file:///I:\HI-FIV\Aim%201\Models\Aim%201c\Literature\Xiao%202010%20Accuracy%20of%20Marginal%20Structural%20Cox%20Model%20Estimators.pdf)). This both forces the average weight to be one and more equally distributes weights among the time periods.

Using a combination of stabilization, truncation, and normalization helped to prevent patient-periods with extreme weights from monopolizing the analyses, set the weighted pseudo-sample to the same size as the unweighted sample, and better balance the inclusion of different time points. Additionally, we chose to only incorporate the first 6 time periods of each patient, though patients had up to 10 periods available. This limited the effects of MSM weight multiplication that can result in excessively large or small weights in late time periods.

The IPTW marginal structural models utilized generalized estimating equations with the following formula (example for Rx Refill/PDC):

PDC ~ Rx Refill + FY Half of Registration + Race/Ethnicity + Age at Entry + Sex + Time Since HIV Diagnosis at Entry + Non-VA Indicator + Baseline Outpatient Visits + Baseline HIV Outpatient + Baseline Inpatient + Baseline Rurality + Baseline ADI + Baseline Housing Status + Baseline Elixhauser + Baseline SUD + Baseline AUD + Baseline Psychoses + Baseline Depression + Baseline Bipolar + Baseline PTSD + Baseline VISN + Baseline PDC + Wave/Period Number of Patient (i.e. time in cohort)

Appendix 4. R version 3.6.1 packages used

| **Package Name** | **Version** | **Usage** |
| --- | --- | --- |
| dplyr | 0.8.3 | Filtering data |
| geepack | 1.2-1 | Execute GEE analyses with weights |
| ggplot2 | 3.2.1 | Make stylized plots |
| parallel (part of base R) | 3.6.1 | Set up cluster for parallelized SuperLearner |
| rms | 5.1-4 | Restricted cubic splines function |
| RODBC | 1.3-16 | Importing data from SQL |
| SuperLearner | 2.0-26 | Create and run parallelized snowSuperLearner models |
| tidyr | 1.0.0 | Convert data from long to wide form |
| xlsx | 0.6.1 | Export data tables to Excel |

Appendix 5. STROBE Checklist

|  | Item No | Recommendation | Page  No |
| --- | --- | --- | --- |
| **Title and abstract** | 1 | (*a*) Indicate the study’s design with a commonly used term in the title or the abstract | 3 |
|  |  | (*b*) Provide in the abstract an informative and balanced summary of what was done and what was found | 3 |
| Introduction | | | |
| Background/rationale | 2 | Explain the scientific background and rationale for the investigation being reported | 5-7 |
| Objectives | 3 | State specific objectives, including any prespecified hypotheses | 6 |
| Methods | | | |
| Study design | 4 | Present key elements of study design early in the paper | 7-9 |
| Setting | 5 | Describe the setting, locations, and relevant dates, including periods of recruitment, exposure, follow-up, and data collection | 7 |
| Participants | 6 | (*a*) *Cohort study*—Give the eligibility criteria, and the sources and methods of selection of participants. Describe methods of follow-up  *Case-control study*—Give the eligibility criteria, and the sources and methods of case ascertainment and control selection. Give the rationale for the choice of cases and controls  *Cross-sectional study*—Give the eligibility criteria, and the sources and methods of selection of participants | N/A |
|  |  | (*b*) *Cohort study*—For matched studies, give matching criteria and number of exposed and unexposed  *Case-control study*—For matched studies, give matching criteria and the number of controls per case | 7 |
| Variables | 7 | Clearly define all outcomes, exposures, predictors, potential confounders, and effect modifiers. Give diagnostic criteria, if applicable | 8-10 |
| Data sources/ measurement | 8* | For each variable of interest, give sources of data and details of methods of assessment (measurement). Describe comparability of assessment methods if there is more than one group | 8-10 |
| Bias | 9 | Describe any efforts to address potential sources of bias | 7, 10 |
| Study size | 10 | Explain how the study size was arrived at | 7-8 |
| Quantitative variables | 11 | Explain how quantitative variables were handled in the analyses. If applicable, describe which groupings were chosen and why | 8-10 |
| Statistical methods | 12 | (*a*) Describe all statistical methods, including those used to control for confounding | 10-11 |
|  |  | (*b*) Describe any methods used to examine subgroups and interactions | 10-11 |
|  |  | (*c*) Explain how missing data were addressed | 9 |
|  |  | (*d*) *Cohort study*—If applicable, explain how loss to follow-up was addressed  *Case-control study*—If applicable, explain how matching of cases and controls was addressed  *Cross-sectional study*—If applicable, describe analytical methods taking account of sampling strategy | N/A |
|  |  | (*e*) Describe any sensitivity analyses |  |

Continued on next page

| Results | | | |
| --- | --- | --- | --- |
| Participants | 13* | (a) Report numbers of individuals at each stage of study—eg numbers potentially eligible, examined for eligibility, confirmed eligible, included in the study, completing follow-up, and analysed | 12 |
|  |  | (b) Give reasons for non-participation at each stage | 12 |
|  |  | (c) Consider use of a flow diagram | 12 |
| Descriptive data | 14* | (a) Give characteristics of study participants (eg demographic, clinical, social) and information on exposures and potential confounders | 21 |
|  |  | (b) Indicate number of participants with missing data for each variable of interest | 21 |
|  |  | (c) *Cohort study*—Summarise follow-up time (eg, average and total amount) | 7-8 |
| Outcome data | 15* | *Cohort study*—Report numbers of outcome events or summary measures over time | 7-8 |
|  |  | *Case-control study—*Report numbers in each exposure category, or summary measures of exposure |  |
|  |  | *Cross-sectional study—*Report numbers of outcome events or summary measures |  |
| Main results | 16 | (*a*) Give unadjusted estimates and, if applicable, confounder-adjusted estimates and their precision (eg, 95% confidence interval). Make clear which confounders were adjusted for and why they were included | 21-25 |
|  |  | (*b*) Report category boundaries when continuous variables were categorized | 10 |
|  |  | (*c*) If relevant, consider translating estimates of relative risk into absolute risk for a meaningful time period | N/A |
| Other analyses | 17 | Report other analyses done—eg analyses of subgroups and interactions, and sensitivity analyses | 13-14 |
| Discussion | | | |
| Key results | 18 | Summarise key results with reference to study objectives | 11-13, 15 |
| Limitations | 19 | Discuss limitations of the study, taking into account sources of potential bias or imprecision. Discuss both direction and magnitude of any potential bias | 16 |
| Interpretation | 20 | Give a cautious overall interpretation of results considering objectives, limitations, multiplicity of analyses, results from similar studies, and other relevant evidence | 15-16 |
| Generalisability | 21 | Discuss the generalisability (external validity) of the study results | 15 |
| Other information | | | |
| Funding | 22 | Give the source of funding and the role of the funders for the present study and, if applicable, for the original study on which the present article is based | 2 |

*Give information separately for cases and controls in case-control studies and, if applicable, for exposed and unexposed groups in cohort and cross-sectional studies.

**Note:** An Explanation and Elaboration article discusses each checklist item and gives methodological background and published examples of transparent reporting. The STROBE checklist is best used in conjunction with this article (freely available on the Web sites of PLoS Medicine at http://www.plosmedicine.org/, Annals of Internal Medicine at http://www.annals.org/, and Epidemiology at http://www.epidem.com/). Information on the STROBE Initiative is available at www.strobe-statement.org.
